# Fidelity of WRF model in simulating heat wave events over India

**DOI:** 10.1038/s41598-024-52541-2

**Published:** 2024-02-01

**Authors:** Priyanshu Gupta, Sunita Verma, Parthasarathi Mukhopadhyay, R. Bhatla, Swagata Payra

**Affiliations:** 1 Institute of Environment and Sustainable Development, Banaras Hindu University, Varanasi, 221005 Uttar Pradesh India; 2 Indian Institute of Tropical Meteorology, Ministry of Earth Sciences, Pune, 411008 Maharashtra India; 3 Department of Geophysics, Institute of Science, Banaras Hindu University, Varanasi, 221005 Uttar Pradesh India; 4 Department of Remote Sensing, Birla Institute of Technology Mesra, Ranchi, 835215 Jharkhand India; 5 DST-Mahamana Centre of Excellence in Climate Change Research, Banaras Hindu University, Varanasi, 221005 Uttar Pradesh India

**Keywords:** Climate sciences, Environmental sciences

## Abstract

The evaluation of Weather Research and Forecasting (WRF) model has been performed for simulating episodic Heat Wave (HW) events of 2015 and 2016 with varied horizontal resolutions of 27 km for the entire India (d01), 9 km for the North West (NW (d02)) and South East (SE (d03)) domain. Study compares the maximum temperature (T_max_) simulated by WRF model, using six different combination of parameterization schemes, with observations from the India Meteorological Department (IMD) during the HW events. Among the six experiments, Exp2 (i.e., combination of WSM6 microphysics (MP) together with radiation parameterization CAM, Yonsei (PBL), NOAH land surface and Grell-3D convective schemes) is found closest to the observations in reproducing the temperature. The model exhibits an uncertainty of ± 2 °C in maximum temperature (T_max_) for both the regions, suggesting regional temperature is influenced by the location and complex orography. Overall, statistical results reveal that the best performance is achieved with Exp2. Further, to understand the dynamics of rising HW intensity, two case studies of HW days along with influencing parameters like T_max_, RH and prevailing wind distribution have been simulated. Model simulated T_max_ during 2015 reaches up to 44 °C in NW and SE part of India. In 2016, HW is more prevailing towards NW, while in SE region T_max_ reaches upto 34–38 °C with high RH (60–85%). The comparative research made it abundantly evident that these episodic events are unique in terms of duration and geographical spread which can be used to assess the WRF performance for future projections of HW.

## Introduction

Weather and climate extremes have become a widely recognized area, it requires further development in climate research field. In recent time, extreme temperature events such as HWs and hot days have become more frequent over the majority of global land areas^1^ and their impact on ecosystem and society are significant. Numerous studies have reported regional changes in extreme heat events^2–4^. Understanding these regional characteristics of extreme temperature is crucial for developing effective adaptation and mitigation strategies. Manifestation of regional cooling/warming in relation to daytime and night time temperature, as they are linked to humidity, cloudiness, soil moisture, and atmospheric circulation pattern have been studied^5^. Further research is necessary to improve our understanding of underlying mechanism driving these events and their regional variations. The development of more advanced modeling techniques and improved observational data can aid in this endeavour.

Projection of HWs depends on the capability of regional and global model to exemplify the appropriate land surface and atmospheric processes^6^. Therefore, understanding the nature and feedback-driving mechanism i.e., soil moisture/snow or circulation pattern of extreme heat events needed a high-resolution model simulation. The regional climate model (RCM) helps to understand the temperature distribution and its variability or even the climatic events, especially extreme heat and cold wave, ^7,8^ were among the first to use regional climate models (RCMs) to study the climate extremes. Over the past year, the numerical models have improved substantially to represent microphysics, large-scale advection, vertical mixing, and convection which govern extreme events over a region and time. Though there exist limited number of model studies that can resolve mesoscale processes substantially^9,10^, still there are areas of uncertainties. These uncertainties and errors in RCM may arise due to inadequacies in different physical parameterization schemes including microphysics, convection, radiation, land surface and planetary boundary layer^11–14^. Effectiveness of the WRF model, along with its different radiation and urban parameterization schemes, when applied at a 0.5 km resolution for forecasting heatwaves in Odisha have been studied^15^.

Moreover, the regional models used to simulate HWs or other extreme occurrences over India are quite sparse and limited. To address this gap, present study introduces a simulation of HWs using a mesoscale model i.e., Weather Research and Forecasting (WRF) version 4.2 in simulating and capturing the extreme temperatures experienced during the HW period of May 22nd to May 30th, 2015 by employing a number of different model configurations. Analysis of different combination of physical parameterization scheme helps to know which configurations are better in simulating maximum temperatures and HW events over particular region. Doing so will help us to isolate the impacts of physical parameterization while minimizing the complication of other factors. This is done following IMD criteria for HWs over NW and SE India. With this, the present study aims to examine the sensitivity to physical schemes in occurences of HW events with fine-scale (9 km) simulation and understand the role of physical processes in simulating extreme heat events. As NW and SE India has experienced rapid increases in summer warm extremes in recent years, we chose two cases of extreme heat events over NW and SE India in 2015 and 2016, to explore the similarities and differences of two events.

Additionally, by incorporating observation of more variables such as Relative Humidity (RH) and wind, an appropriate combination of WRF physical parametrization configuration is selected that accurately simulates HW events, particularly in the NW and SE India regions. Subsequently, using the optimal combination of parameterization schemes, a long-term simulation (2006–2016) of maximum temperatures (T_max_) is conducted from the 8th–31st May for climatology analysis of HW days. Lastly, following IMD criteria, HW days and their dynamics have been identified for two specific episodes: HW 2015 (22nd–30th May) and HW 2016 (14th–23rd May), using IMD observations and WRF simulated datasets.

This paper is structured as follows: Section "Datasets and methodology" describes the configuration of the WRF experiments and the observational dataset used to evaluate the model performance. Section "Results and discussion" presents the results of performance evaluation. Finally, Section "Conclusions" summarizes the paper conclusions.

## Datasets and methodology

### Description of weather research and forecasting (WRF) model

WRF is a widely used RCM, developed by the National Center for Atmospheric Research (NCAR), the National Oceanic and Atmospheric Administration (NOAA), and other communities. It is a mesoscale model, capable to simulate a wide range of atmospheric phenomena, including clouds, precipitation, winds, and temperature at a resolution ranging from few kilometers to a few hundred meters^16^. It is a numerical based model designed for both weather predictions and research, is configured into three system featuring WPS, WRF and ARW Post^17^. Present paper describes in detail how each process has been included in WRF. It incorporates both physics and dynamics component to simulate the complex interaction between the atmosphere, land surface, and ocean. WRF model have different sets of parameterization configuration for physical process hence it is suitable for a multiphysics approach^18–21^. One of the essential steps in numerical weather simulation is to choose the best sets of physics option for different weather system over various geographical region and time duration. Before long-term integration, sensitivity assessment of the model to different dynamical configurations and physical parameterization is one of the vital steps^22^. In this respect WRF model is a convenient tool as it provides plenty of schemes. In simulating the relevant climatic variables, e.g., temperature and precipitation, it is highly dependent on various physical process like; cumulus convection, planetary boundary layer (PBL), land surface, and microphysical processes^23–25^. Numerous studies have been undertaken with the WRF model to select the most suitable configuration for regional climate simulations^26–28^. WRF model capability to simulate very significant severe temperature events during HW days towards coastal regions of India. evaluated the unforeseen HW conditions over SE coastal India in May, 2015^29^. Tropospheric temperature, upper levels anticyclonic flow, higher outgoing longwave radiation, below normal precipitable water as a major synoptic feature associated with HW occurrence^30^.

### General model set up and simulations

**Figure 1:**
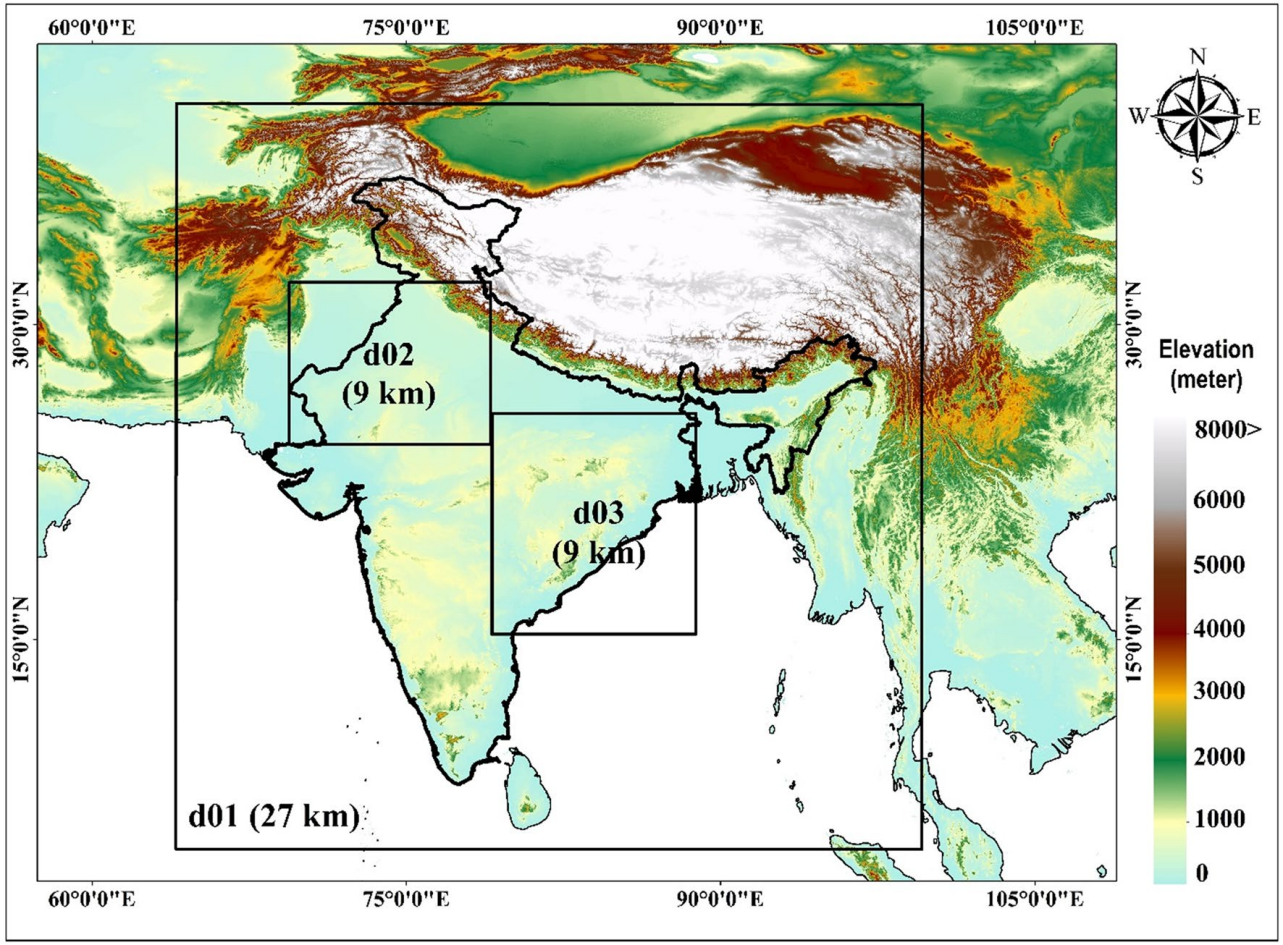
Selected domains for WRF simulation over India.

Present study utilizes Advanced Research (ARW) version 4.2 of WRF, a fully compressible and nonhydrostatic model. The WRFv4.2 offers significant advantages compared to its previous version for HW studies. It incorporates improved physical parameterizations that better represents the atmospheric process relevant to HWs, such as land surface process, boundary layer turbulence, and radiation. This leads to more accurate simulations of HW characteristics, including intensity, duration, and spatial extent. Additionally, WRFv4.2 feature enhanced computational efficiency and scalability, allowing for higher resolution simulations and the ability to capture localized HW phenomena with greater precision. The initial and lateral boundary condition for the simulation is carried out with NCEP (National Centres for Environmental Prediction) FNL reanalysis meteorological data archived at 1° × 1° grid. The boundary condition data includes the land use (USGS-24 category terrestrial; LULC-30s data), terrain elevation-2 min and vegetation fraction-10 min data. Terrain and vegetation fraction datasets are available from the standard WRF pre-processing System (WPS) v4.2. High-resolution experiments using two nested domains with horizontal resolutions of 27 km and 9 km have been used for the study is represented in Fig. 1. Three domains are selected i.e., mother domain, d01 (27 km) and daughter domains; d02 (9 km) and d03 (9 km). The detailed model descriptions and configurations are presented in Table 1. The experiment is specially performed on HW affected India regions i.e., NW (d02) and SE (d03). It provides a balanced and comprehensive approach to study the heatwave dynamics, considering their distinct climatic conditions, geographical features, and societal vulnerabilities. Domain 1 (d01) covers entire India (6–38°N and 65–98°E) and nested domain (two-way nesting) d02 extend from (24.25–31.4°N and 69.7–78.4°E) covering some portion of states like Rajasthan, Punjab, Haryana, Madhya Pradesh and domain d03 extend from (15.3–25.75°N and 79.18–88.7°E) covering Jharkhand, Odisha, Andhra Pradesh, Telangana etc. The detailed model configurations are shown in Table 1.

**Table 1:** Description of model configuration.

Model version	4.2
Dynamics	Non-Hydrostatic
Map Projection	Mercator
e_we	133, 100, 109
e_sn	143, 94, 127
Horizontal and spatial resolution	27 km, 9 km, 9 km
Horizontal grid system	Arakawa grid staggering
Time integration scheme	Runge–Kutta 3rd order
Vertical coordinate	Terrain-following hybrid (sigma-pressure) (50 sigma levels) vertical coordinate
Initial and boundary conditions	NCEP Final Analysis (FNL) reanalysis

### Sensitivity analysis and physics schemes used.

WRF provides a number of physical configuration choices, allowing the model to be optimised for a certain weather phenomenon and location. The parameterization approaches are chosen based on earlier studies taken from different papers^31,32^. In this study six experiments considering different combination of the microphysics (MP; four schemes), planetary boundary layer (PBL; two schemes), surface layer (three schemes), shortwave and longwave radiation (RA; three schemes) and of convection (CU; two schemes) scheme are evaluated. The study implemented six distinct physical parameterization schemes, which are extensively discussed in Table 2.

**Table 2:** Different combination of physics schemes used for maximum temperature during heat wave events.

Simulation name	Microphysics (MP)	Radiation (RA)	Land surface		Boundary layer (PBL)	Cumulus convection (CU)	Exp- code
			sf-sfclay	sf-surface			
Exp1	Lin (2)	-	MYJ-ETA (2)	NOAH (2)	MYJ (2)	Kain-Fritsch (1)	22,221
Exp2	WRF-SM6 (6)	CAM (3)	Revised MM5 (1)	NOAH (2)	Yonsei (1)	Grell-3D (5)	631,215
Exp3	Thomson (8)	RRTM (1)	ETA (2)	NOAH (2)	Mellor-yamada janic (2)	Kain-Fritsch (1)	812,221
Exp4	WRF-SM6 (6)	RRTM (1)	MM5 (91)	RUC land surfaces (3)	Yonsei (1)	Grell 3D (5)	6,191,315
Exp5	WSM5 (4)	RRTMG (4)	Revised MM5 (1)	NOAH (2)	Yonsei (1)	Kain-Fritsch (1)	441,211
Exp6	WRF-SM6(6)	CAM (3)	Revised MM5 (1)	1 (5layer thermal diffusion)	Yonsei (1)	Kain-Fritsch (1)	631,111

In order to know its impact on the T_max_, RH and wind speed. initially multiple WRF simulations with different physical parameterizations have been conducted. From these sets of simulation, we have identified a reduce sets of six simulation that best represents the HWs observed in 2015. Subsequently, six simulations are conducted during HW duration of 19th–30th May 2015, with a spin-up time of 72 h. Afterward, based on robust statistical measures such as fractional bias (FB), Normalized mean square error (NMSE), Root mean square error (RMSE), correlation coefficient (r), and degree of index (d), the most optimal configuration is chosen to simulate another HW event occurring from 8th to 31st May, 2016. The selected configuration shows best score for all the variables based on initial test.

### Datasets used

To evaluate the best performing simulations, IMD observed gridded (0.5° × 0.5°) maximum temperature datasets have been used. These datasets are spatially interpolated, providing estimates of maximum temperature values across a regular grid point covering India. IMD datasets have been created using a statistical interpolation method to estimate the temperature values at grid points based on the observed values from a network of weather stations across the country. To match the spatial resolution, WRF data at 27 km is extrapolated to 50 km of IMD observed datasets. Further, with the station-based observations over India, a closer inspection of model performance is indeed mandatory to know the variability and pattern of climate behaviour. Further, to validate the d02 (Delhi, Jaipur) and d03 (Ranchi, Rourkela) domains, T_max_, RH, and wind speed data has been obtained from the METAR datasets available at https://www.wunderground.com. The METAR data obtained from wunderground provides detailed weather observations of a specific location. It includes information such as temperature, dew point, humidity, wind speed and direction, atmospheric pressure, precipitation, and other relevant meteorological parameters^31,33–35^.

Additionally, in order to examine the dynamics, various meteorological parameters have been assessed using ERA5 dataset (as shown in the supplementary section). Moreover, HW days has been identified using long term climatology of WRF simulations (2006–2014) and IMD observed (1986–2014). Study by^36^ utilized a 9-year WRF climatology to predict the Indian summer monsoon during active and break periods.

### Methodology

#### Descriptive statistics

Uncertainty in model datasets have been analysed by robust statistics. It comprises the Normalized mean square error (NMSE), Root mean square error (RMSE), Fractional bias (FB), correlation coefficient (r), Index of agreement (d) and fraction of model predictions within a factor of two of observation (FA2) are explain by following equations.

1 $$\mathbf{N}\mathbf{M}\mathbf{S}\mathbf{E}=\frac{{\stackrel{-}{\left({\mathbf{X}}_{\mathbf{M}}-{\mathbf{X}}_{\mathbf{O}}\right)}}^{2}}{{\overline{\mathbf{X}} }_{\mathbf{M}}{\overline{\mathbf{X}} }_{\mathbf{O}}}$$

2 $$\mathbf{F}\mathbf{B}=\frac{\left({\overline{\mathbf{X}} }_{\mathbf{M}}-{\overline{\mathbf{X}} }_{\mathbf{O}}\right)}{0.5\left({\overline{\mathbf{X}} }_{\mathbf{M}}+{\overline{\mathbf{X}} }_{\mathbf{O}}\right)}$$

3 $$\mathbf{R}=\frac{\stackrel{-}{\left({\mathbf{X}}_{\mathbf{O}}-{\overline{\mathbf{X}} }_{\mathbf{O}}\right)\left({\mathbf{X}}_{\mathbf{M}}-{\overline{\mathbf{X}} }_{\mathbf{M}}\right)}}{{{\varvec{\upsigma}}}_{{\mathbf{X}}_{\mathbf{M}}}{{\varvec{\upsigma}}}_{{\mathbf{X}}_{\mathbf{O}}}}$$

4 $$({\text{FA}}2 =\mathrm{ Fraction of data which satisfy})\to 0.5\le \frac{{\mathbf{X}}_{\mathbf{M}}}{{\mathbf{X}}_{0}}\le 2.0$$

5 $$\mathbf{d}=1-\frac{{\sum }_{\mathbf{i}=1}^{\mathbf{n}}{\left({\mathbf{X}}_{{\mathbf{O}}_{\mathbf{i}}}-{\mathbf{X}}_{{\mathbf{M}}_{\mathbf{i}}}\right)}^{2}}{{\sum }_{\mathbf{i}=1}^{\mathbf{n}}{\left(\left|{\mathbf{X}}_{{\mathbf{M}}_{\mathbf{i}}}-{\overline{\mathbf{X}} }_{\mathbf{O}}\right|+\left|{\mathbf{X}}_{{\mathbf{O}}_{\mathbf{i}}}-{\overline{\mathbf{X}} }_{\mathbf{O}}\right|\right)}^{2}}$$

where, X_M_: Model; X_O_: observations; X̅_O_ and X̅_M_: average of respective dataset; ${\upsigma }_{{{\text{X}}}_{{\text{M}}}}\mathrm{ and }{\upsigma }_{{{\text{X}}}_{{\text{O}}}}$: standard deviation of model and observation datasets.

#### Taylor diagram

A Taylor diagram is a graphical tool used to assess the accuracy and reliability of climate or weather model simulations by comparing them with observation (Taylor, 2001). In essence, the Taylor diagram provides a means to assess the similarity of model simulations to observed data, taking into account various statistical metrics. By comparing the correlation coefficient and RMS differences of the model and observation, as well as their respective variances, the diagram offers insight into the accuracy and reliability of the model.

The mathematical formula for the Taylor diagram is expressed as follows:

$$E^\prime 2 = \sigma f2 + \sigma r2 + 2\sigma f\sigma r\rho$$

where E' is the centered root-mean-square difference between the model and observation, σf and σr represents the variances of the model simulations and observation, respectively, and ρ denotes the correlation coefficient between the model and observation.

Further, to identify the HW events the present study adopted following IMD criteria as shown in Table 3:

**Table 3:** IMD criteria for Heat wave analysis.

Region	Normal maximum temperature	Deviation from Normal temperature	Conditions
Plain area	≤ 40 °C	5 °C to 6 °C	Heat wave
	> 40 °C	4 °C to 5 °C	Heat wave
Coastal area	≥ 37 °C	≥ 4.5 °C	Heat wave

When the actual maximum temperature persists at 45 °C or above; Heat wave should be declared irrespective of the normal maximum temperature.

During a heatwave period in India, as reported by the India Meteorological Department (IMD) or other government agencies, synoptic features typically include sustained high temperatures exceeding defined thresholds, often accompanied by the dominance of a strong anticyclone fostering subsidence and clear skies. Low humidity levels contribute to arid conditions, and stable atmospheric conditions with an inversion layer impede vertical mixing, trapping heat near the surface. Reduced wind speeds and prolonged sunshine further exacerbate the high temperatures.

## Results and discussion

First, six simulations using WRF model has been conducted during HW duration of 19th–30th May, 2015 considering 72 h spin up time. The WRF model is run at 27 km with a sub-domain of 9 km. The initial and boundary conditions for the simulations is derive from the NCEP FNL dataset (http://rda.ucar.edu/datasets/ds083.2/), which has a time resolution of 6 h and a spatial resolution of approximately 1° × 1° (111 km × 111 km).

### Model simulations and statistical evaluation

**Figure 2:**
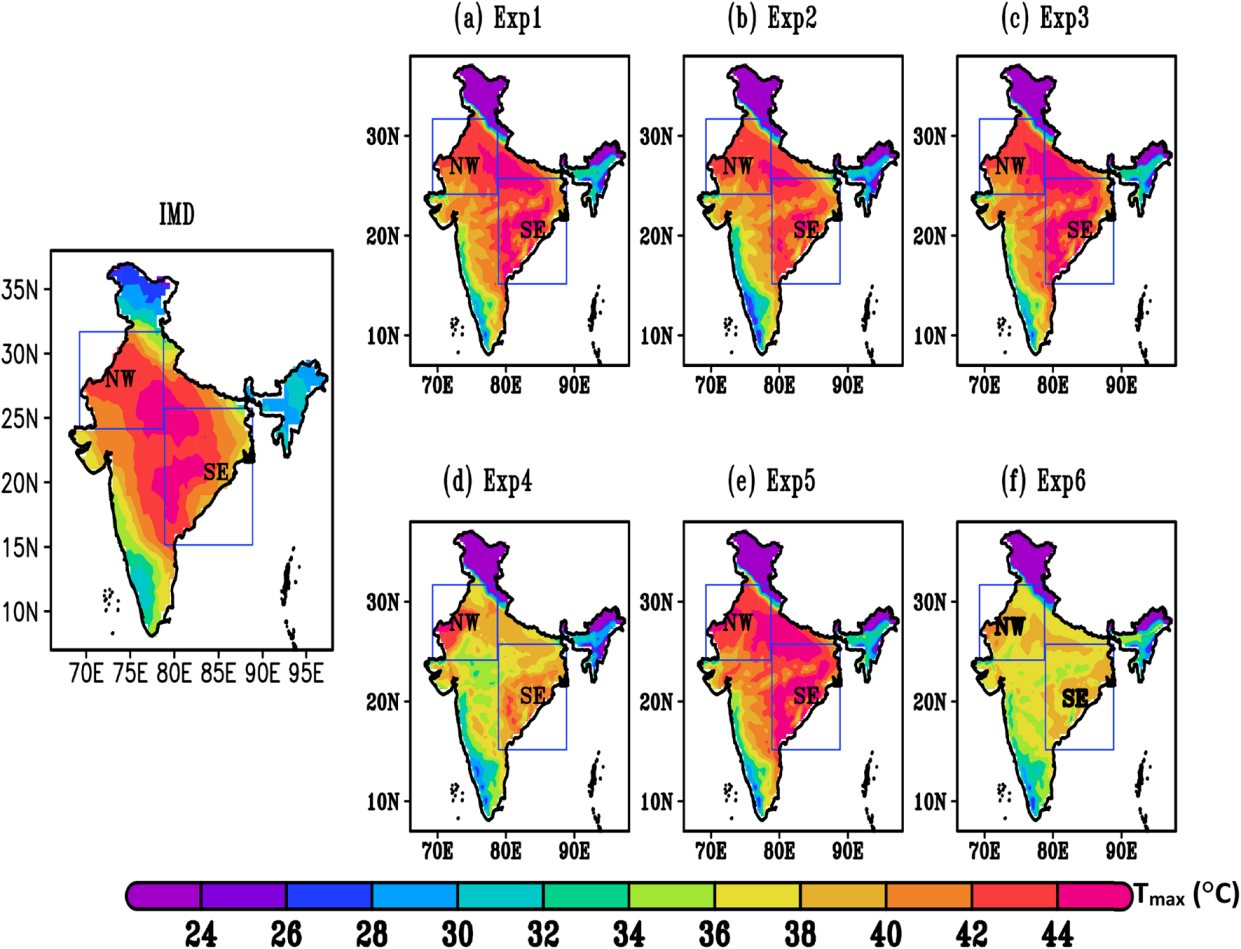
(**a**–**g**). Daily average all India maximum temperature (°C) during HW events (22nd–30th, May) (**a**) IMD gridded data set (**b**) Exp1, (**c**) Exp2, (**d**) Exp3, (**e**) Exp4, (**f**) Exp5, and (**g**) Exp6. Blue box represents NW and SE India i.e., the major HW affected sub regions of the India.

Spatial distribution and variability of IMD observed T_max_ and corresponding model values during HW duration of 22nd–30th May, 2015 are shown in Fig. 2. A remarkable warming over entire India is shown with high temperature value of > 44 °C in NW, SE and central regions of around 15°N–30°N latitude. The highest temperature up to 44 °C is recorded over NW as well as SE India, whereas the lowest temperature of about 28–30 °C is recorded over the Western Himalaya and North-East region. Origination of HW and high temperature over India are likely sourced from Thar desert and Rajasthan^37^. Prevailing dry continental westerlies, lack of vegetation, subsidence and abundance of dust particles are major contributing factors for high temperature over Rajasthan and Thar Desert^38^. Further, WRF simulation for T_max_ using different combination of parameterization schemes (as shown in Table 2) are compared with IMD observed datasets. The T_max_ variability during HW event is well captured (24–44 °C) by Exp1, Exp2, Exp3 of and Exp5 against observed T_max_ (28–44 °C) with slight overestimation. Model capability is however good in capturing the T_max_ over the Indian regions except Western Himalaya. A few earlier studies, have also observed the tendency of WRF to cause lower temperatures across the Himalaya region^39^. Although the actual causes are yet unknown, they are likely linked to the cloud radiative process. However, for Exp4 and Exp6 the model is unable to perform well (− 5 °C of underestimation). This combination of configuration is not up to confidence level to consider for further study. This might be due to land surface schemes which play major role in simulating HW events.

**Figure 3:**
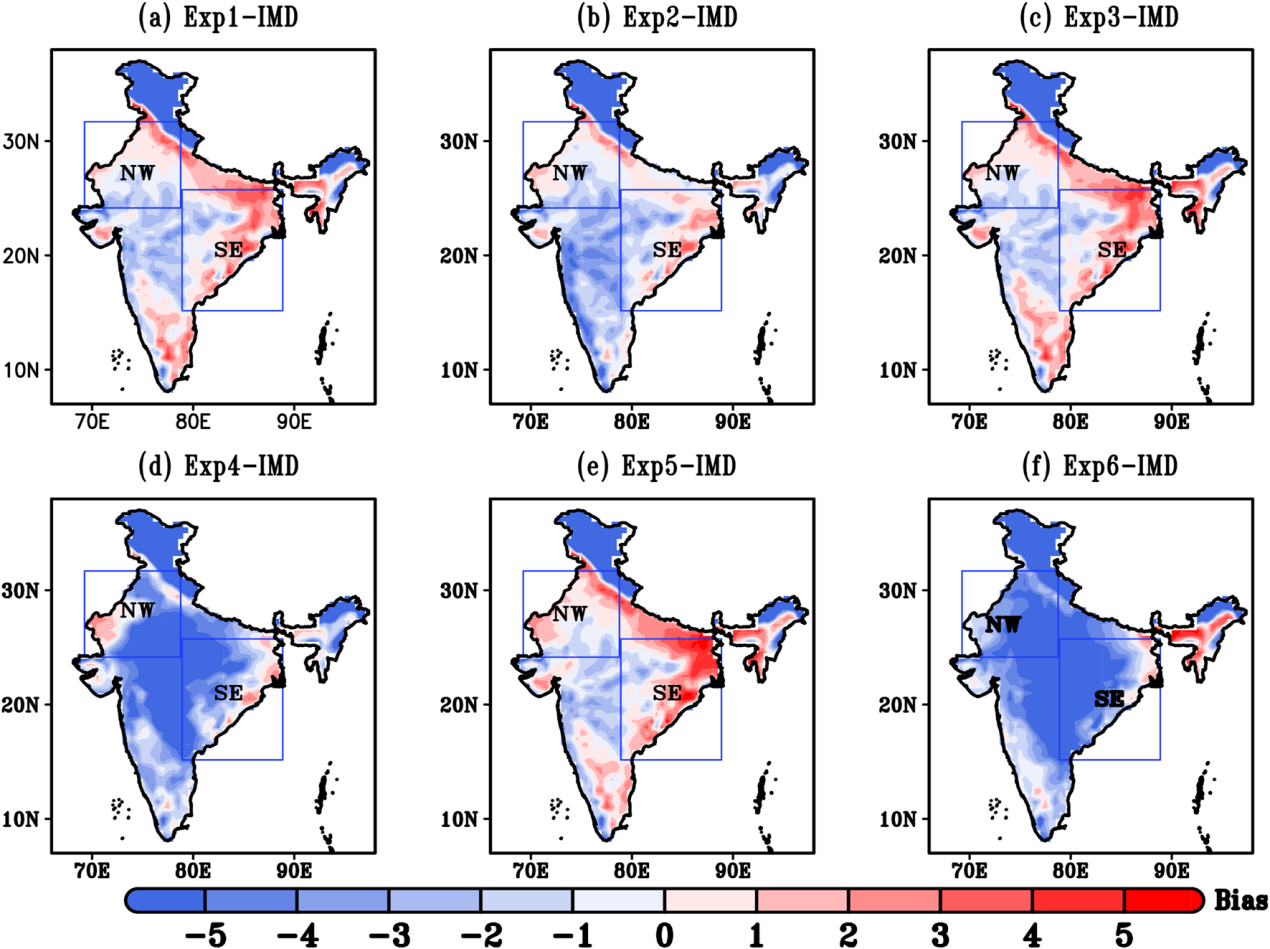
(**a**–**f**) Spatial distribution of maximum temperature bias (°C) during HW event (22nd–30th may, 2015) (**a**) Exp1 minus IMD, (**b**) Exp2 minus IMD, (**c**) Exp3 minus IMD, (**d**) Exp4 minus IMD, (**e**) Exp5 minus IMD, (**f**) Exp6 minus IMD.

**Figure 4:**
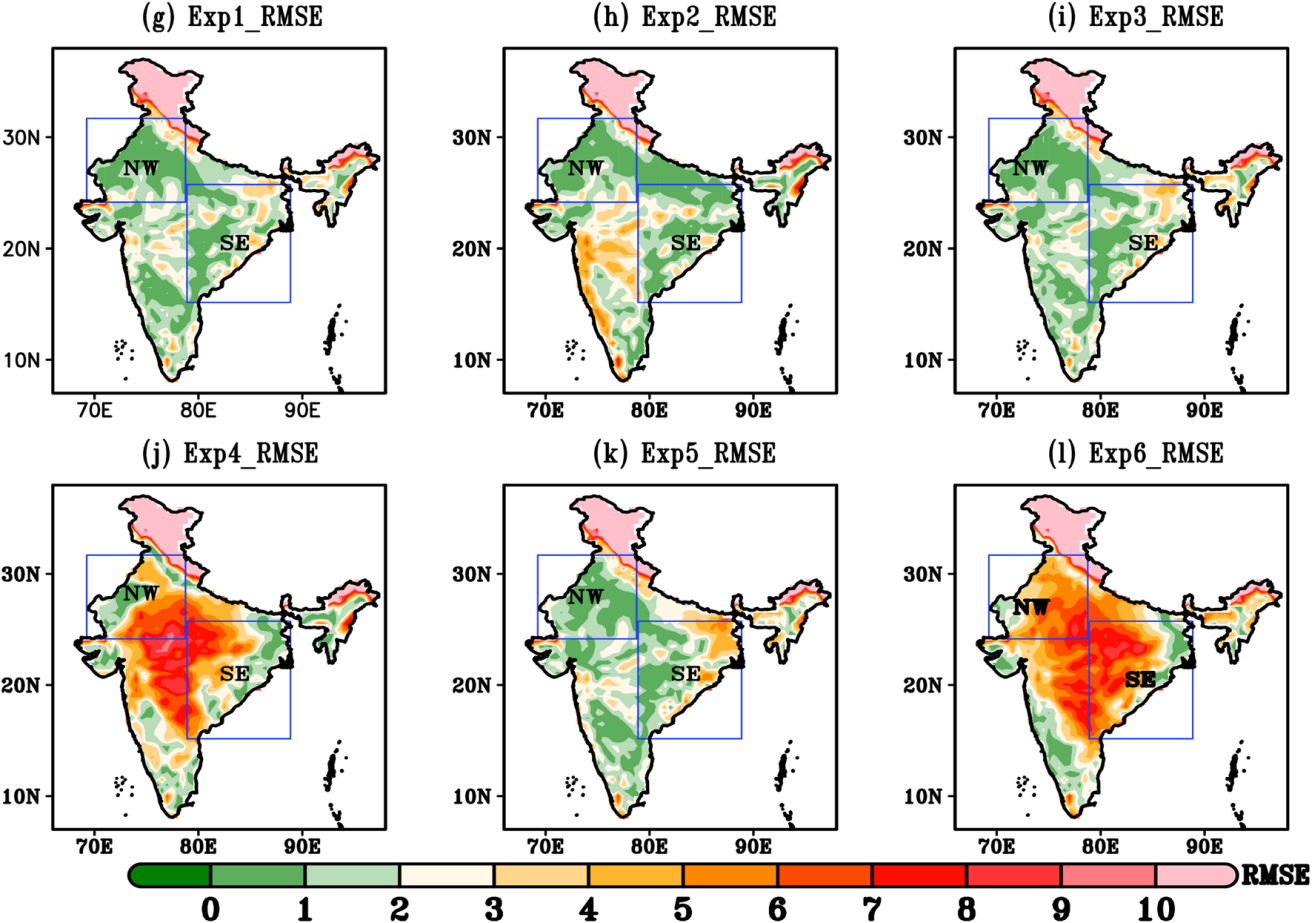
(**g**–**l**). Spatial RMSE during HW days (22^nd^–30^th^ May,2015) (**g**) Exp1, (**h**) Exp2, (**i**) Exp3, (**j**) Exp4, (**k**) Exp5 and (**l**) Exp6.

Daily mean T_max _bias during 22nd–30th May 2015 is shown in Fig. 3. The bias pattern in different combination of experiments present noticeable differences over most part of the domain. It is seen that all model simulation except Exp4 and Exp6, overestimate the T_max_ around NW and SE India by ± 1 °C and ± 2 °C, respectively. Basically, Exp2 exhibit the least biases (± 1 °C) in describing the T_max_ against the observational data relative to the other Experiments. Thus, Exp2 which is combination of WSM6 (microphysics schemes), Yonsei (PBL schemes), NOAH (land surface scheme) with Grell-3D convective parameterization schemes outperforms other Experiments for the maximum temperature fields. The underestimation of T_max_ (< 5 °C) in Exp4 and Exp6 over the hilly region might be due to large number of uncertainties present in climate data, which is mostly in the central and mountainous areas. Overall, Exp2 is able to simulate the spatial pattern of summer T_max_ (24–44 °C) which is closer to the observations (28–44 °C). In general, the simulation of Exp2 shows a smaller temperature bias compared to other experiments and effectively captures the temperature range associated with HWs, as observed. Furthermore, Fig. 4g–l presents the spatial root mean square error (RMSE) statistics for the six different simulations conducted with WRF. RMSE pattern reveals the short-term performances of a model by comparison with observed datasets. A smaller value indicates the better model performance. For all the combination of Experiments, RMSE ranges between 0 °C–10 °C. Exp2 describes a best performing simulation with low RMSE values. The magnitude of RMSE over parts of NW and SE India is in order of 0–5 °C. Exp4 and Exp6 shows higher RMSE (6–10 °C) values specially in central, NW and SE regions.

### Descriptive statistics of station datasets

With the station-based observations over India, a closer inspection of model performance is indeed mandatory to know the variability and pattern of climate behaviour. In Table 4 model performance for NW(d02) and SE(d03) domain have been examined using different statistics e.g., NMSE, RMSE, FB, R and d to estimate the linked biases and errors. NMSE is use to evaluate the maximum temperature and RH datasets of model. It avoids bias towards model that over predict or under predict and to emphasize the scatter in the entire datasets of sample concentrations^40^. Exp2 reveals low NMSE, RMSE and FB value for temperature and RH datasets and indicates good simulation of this combination of model. For Exp2 a low NMSE value of 0.02 (Delhi), 0.03 (Jaipur) of d02 domain and 0.1(Ranchi), 0.05 (Rourkela) of d03 domain is observed. Further FB has been used to determine the overestimation or underestimation of model datasets. For the combination of Exp2, a low FB value is also seen in Delhi (0.006), Jaipur (0.0067), Ranchi (0.02) and Rourkela (0.0004) cities where model undergoes overestimation w.r.t observed datasets. For Exp2, there is a strong correlation among all the cities, with r values of 0.97, 0.95, 0.96, and 0.90 for Delhi, Jaipur, Ranchi, and Rourkela cities, respectively. Additionally, the degree of agreement between the model and observed datasets is determined by calculating the d value. Exp2 exhibits large d values (0.8–0.9), indicating a high level of agreement between both datasets.

**Table 4:** T_max_ (°C) and RH (%) Normalized mean square error (NMSE), root mean square error (RMSE), FB, r and d values for May, 2015 HW days for d02 and d03 selected stations.

Regions	Stations		T_max_ (°C)					RH (%)				
			NMSE	RMSE	FB	R	d	NMSE	RMSE	FB	R	d
NW	Delhi	Exp1	0.0022	1.79	0.0103	0.93	0.96	0.3	10.04	0.034	0.47	0.68
		Exp2	0.0017*	1.58*	0.006*	0.94*	0.97*	0.28*	9.29*	0.123	0.5	0.69*
		Exp3	0.0023	1.84	0.012	0.93	0.96	0.45	10.31	0.37	0.47	0.61
		Exp4	0.014	4.25	− 0.091	0.89	0.85	0.87	24.7	− 0.68	0.64*	0.58
		Exp5	0.004	2.32	0.041	0.93	0.93	0.44	9.98	0.41	0.57	0.67
		Exp6	0.029	5.97	− 0.15	0.85	0.74	1.52	37.97	− 0.94	0.57	0.51
	**Jaipur**	Exp1	0.0024	1.84	0.011	0.94	0.95	0.19	9.5	− 0.19	0.8	0.84
		Exp2	0.0013*	1.39*	0.0067*	0.94*	0.97*	0.12*	6.7*	0.008*	0.82	0.9*
		Exp3	0.0023	1.82	0.0081	0.93	0.95	0.17	8.9	− 0.16	0.81	0.85
		Exp4	0.019	4.9	− 0.113	0.87	0.78	0.97	28.89	− 0.75	0.61	0.57
		Exp5	0.0018	1.61	0.016	0.94*	0.96	0.123	7.24	− 0.09	0.83*	0.89
		Exp6	0.02	5.03	− 0.128	0.86	0.74	1.23	35.09	− 0.88	0.67	0.55
**SE**	**Ranchi**	Exp1	0.012	3.86	0.06	0.79	0.85	1.56	30.39	− 0.92	0.517	0.52
		Exp2	0.012*	3.77*	0.02*	0.75	0.86*	0.112*	13.96*	0.079*	**0.7302**	0.84*
		Exp3	0.012	3.9	0.066	0.8	0.85	1.621	30.65	0.55	0.507	0.53
		Exp4	0.017	4.2	− 0.089	0.81*	0.8	0.798	26.92	− 1.089	0.57	0.68
		Exp5	0.015	4.3	0.078	0.78	0.82	2.39	33.66	0.198	0.34	0.49
		Exp6	0.23	4.8	− 0.11	0.79	0.76	0.174	18.43	0.198	0.61	0.73
	Rourkela	Exp1	0.005	2.79	0.0172	0.94	0.94	0.421	15.54	− 0.4	0.8	0.81
		Exp2	0.0048*	2.69*	0.0004*	0.93	0.94	0.09*	9.23*	0.089*	0.82*	0.89*
		Exp3	0.005	2.77	0.02	0.94*	0.94*	0.46	16.32	− 0.39	0.79	0.79
		Exp4	0.0098	3.67	− 0.083	0.92	0.87	0.526	18.078	− 0.323	0.77	0.78
		Exp5	0.005	2.85	0.041	0.93	0.93	0.51	28.205	0.57	0.61	0.55
		Exp6	0.031	6.29	− 0.17	0.91	0.71	0.74	17.93	0.65	0.77	0.73

The bold values inducating the best performance of the particular experiments with low RMSE, low NMSE, low FB and high r and d values.

**Figure 5:**
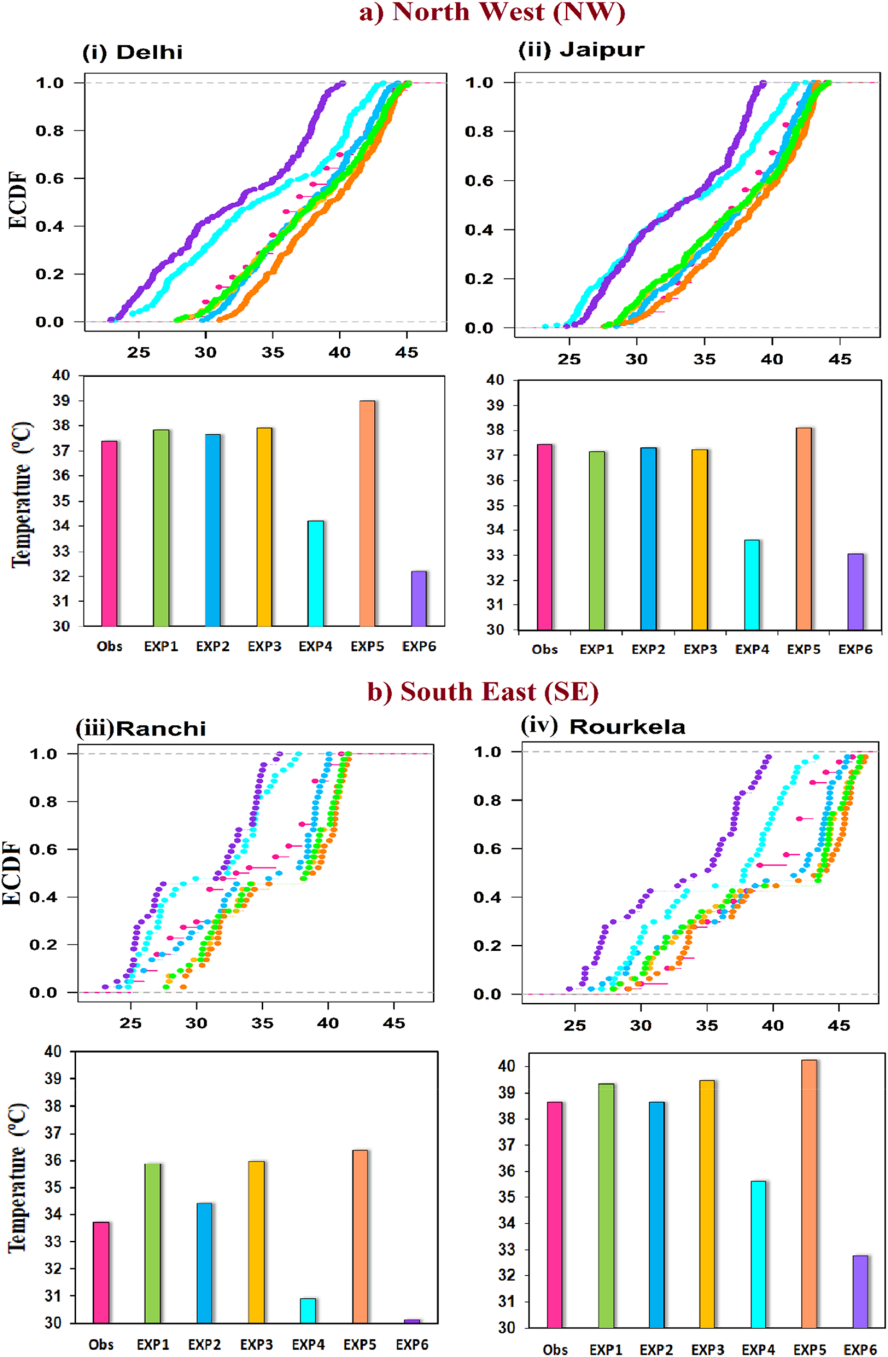
The empirical cumulative distribution function (ECDF) and column plot of hourly datasets of d02 and d03 stations during heat wave events (22nd–30th May, 2015).

**Figure 6:**
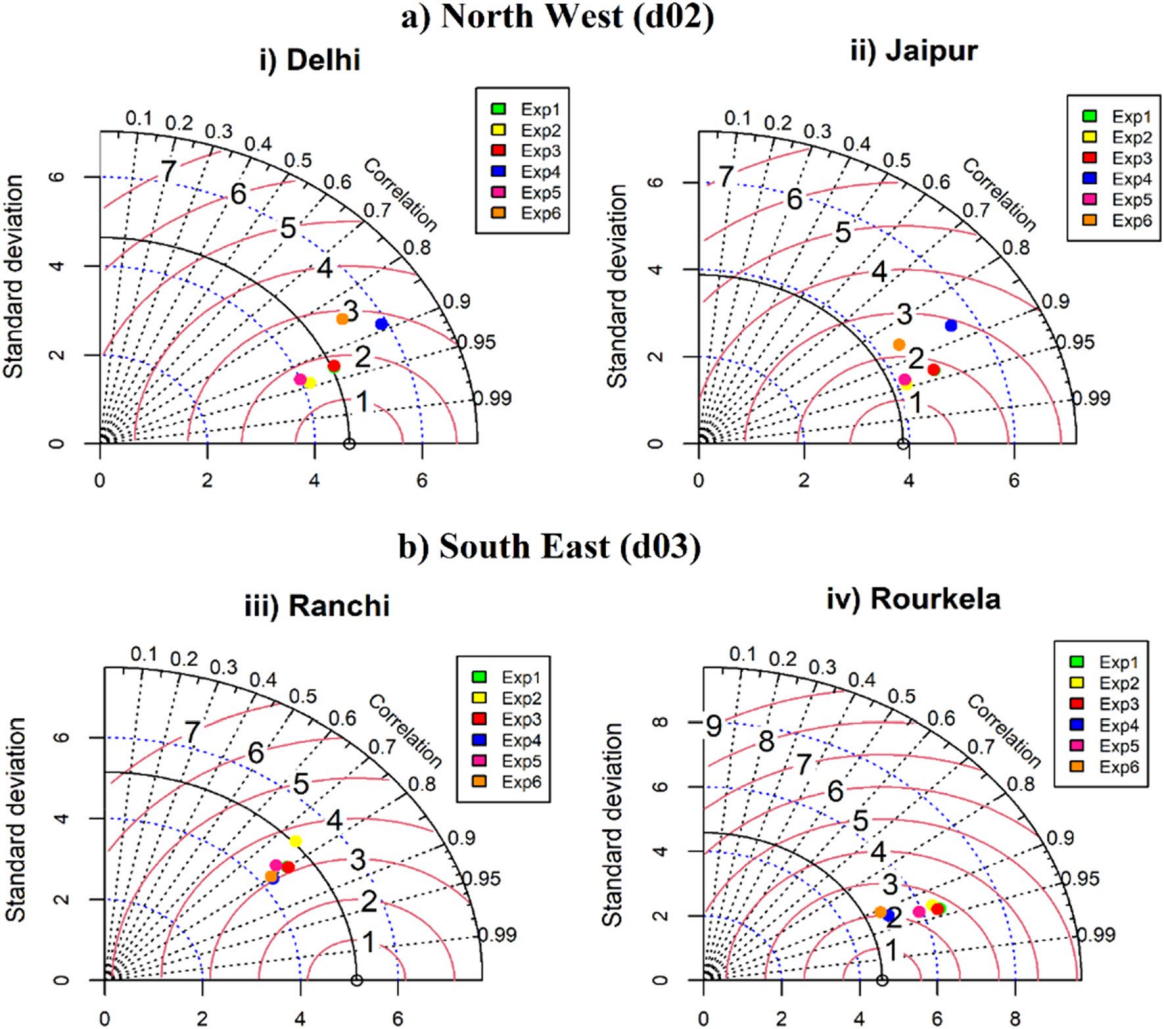
Taylor diagram represents a statistical comparison between observations and six different combinations of WRF simulated maximum temperature (°C) datasets across the Delhi, Jaipur, Ranchi, and Rourkela cities.

Furthermore, empirical cumulative distribution function (ECDF) plot (Fig. 5) has been used to verify the simulated T_max_. In this plot, the data is distributed from lowest to highest values, and a best-fitted distribution line is displayed. If the fitted lines of the observed and modeled data closely align, it indicates a good fit of the data to the distribution. The ECDF values of Exp2 and Exp1 demonstrate close proximity to the observation line across all stations within the d02 (Delhi, Jaipur) and d03 (Ranchi, Rourkela) domains, as depicted in Fig. 5ai–ii,biii–iv. While Exp4 and Exp6 does not match closely with observation. Further, column plot also represents the similar results such as simulated T_max_ of Exp2 are closer to the observation over Delhi, Jaipur, Ranchi and Varanasi stations. Due to missing no. of observed data in Ranchi and Rourkela station, the data seems to be sparsely distributed (Fig. 5ii). In this work simulation of WRF is crucially dependent on land surface schemes. The performance of NOAH land surface in Exp1, Exp2, Exp3 and Exp5 are overestimating the observed temperature by 2–3 °C while RUC land surface and thermal diffusion, surface clay used in Exp4 and Exp6 underestimated by − 4 to − 5 °C against the observation in NW and SE regions. The meteorological evaluation (T_max_) of the WRF simulated six experiments are compared with observed datasets is represented by Taylor diagram in Fig. 6i–ii. In Fig. 6, multiple aspects of complex model are evaluated in terms of correlation, standard deviation (SD) and centred root-mean square (RMS) difference. Statistics for six experiments were computed and among all configurations, Exp2 performs better especially over Delhi and Jaipur cities of d02 domain. In Delhi city, the correlation with observations is about 0.95 with centred RMS value of 1.3 and standard deviation is 4.0 i.e., less than observed SD (4.3) shown by the solid arc. Jaipur city also indicates best performance of Exp2 simulation with high correlation of 0.95 and low SD (4.1) and RMS (1.5) values. Though in Fig. 6i,ii it can be seen that Exp2 and Exp5 generally agree best with observations, each with almost same correlation and SD. Exp2, however, has a slightly lower RMS than Exp5 over both the stations. Further, statics of Ranchi and Rourkela stations of d03 domain have been calculated. In these stations all the configurations are clustered together, indicating similar performance for simulating the maximum temperature. Comparing all the cluster experiments, Exp4 performing slightly better than others.

**Figure 7:**
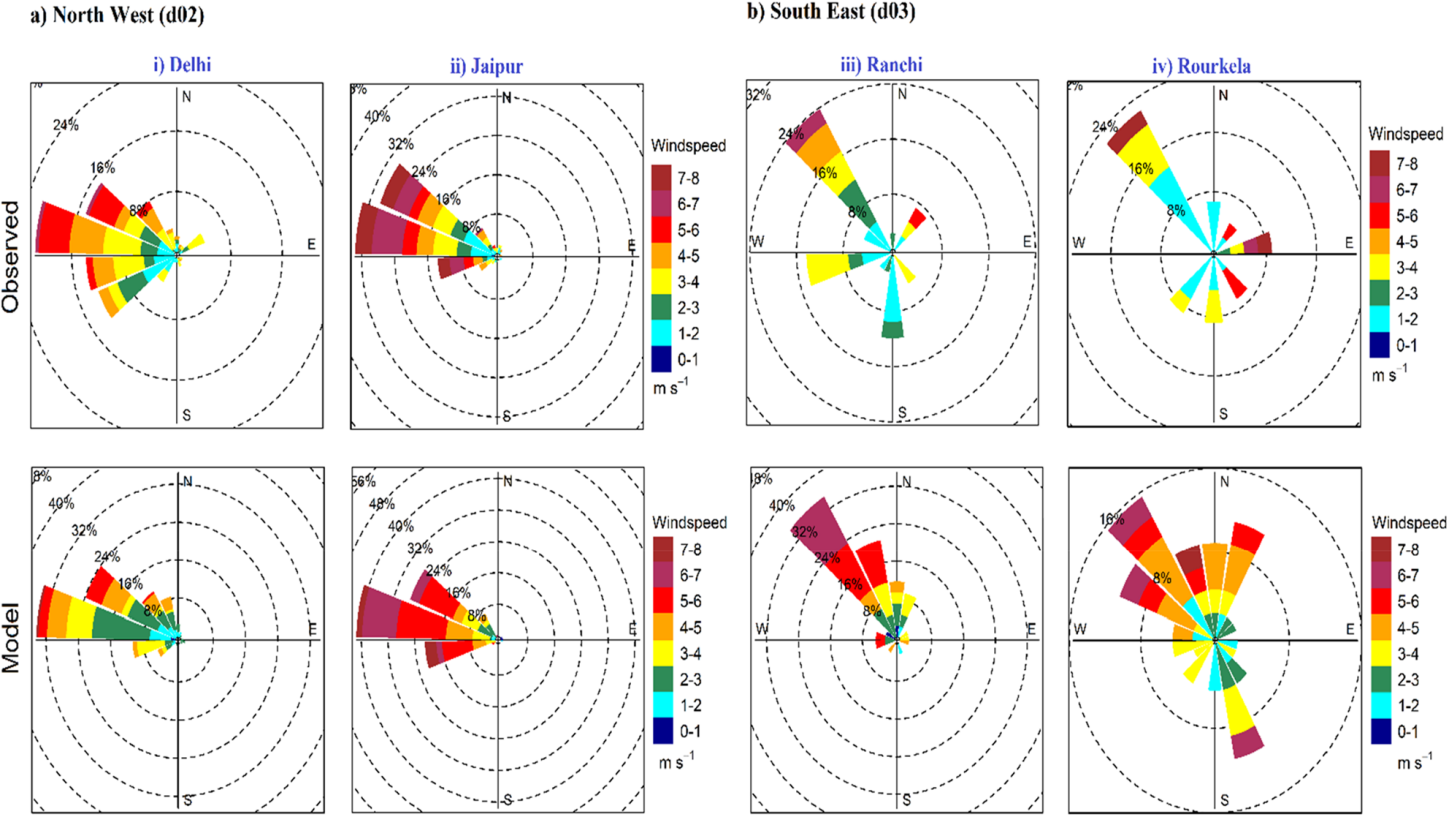
Comparison of wind roses obtained from best performing configuration of Exp2 over (**i**) Delhi, (**ii**) Jaipur, (**iii**) Ranchi and (**iv**) Rourkela with observations, during heat wave period of 22nd–30th May, 2015.

Other meteorological parameters like model simulated wind speed and wind direction at 10m height (u10, v10) in d02 and d03 domain is also compared with the ground-based observation during HW period of 2015 (Fig. 7i–iv). Wind data for best combination of configuration (Exp2) is represented by wind rose plot which characterise wind speed and direction at selected stations. In d02 domain (Delhi and Jaipur) the WRF model simulate most of the prevailing strong winds in west direction, which agrees well with the ground-based observation. In d03 domain (Ranchi and Rourkela) model reproduces predominantly northwest winds.

### Spatiotemporal variation of Heat wave events

A temporarily rise in maximum temperature due to deviation from normal circulation pattern leading to a HW events; it may also be accompanied by high humidity. Case studies are the most efficient method to evaluate the model performance and estimate the occurrence of extreme Heat events. Present study simulates the episodic event of HW 2015 and HW 2016 at 9 km of resolution for NW and SE Indian regions. Using IMD criteria HW days have been analysed by both IMD observed and WRF model datasets.

#### Episodic event of 2015 HW days

**Figure 8:**
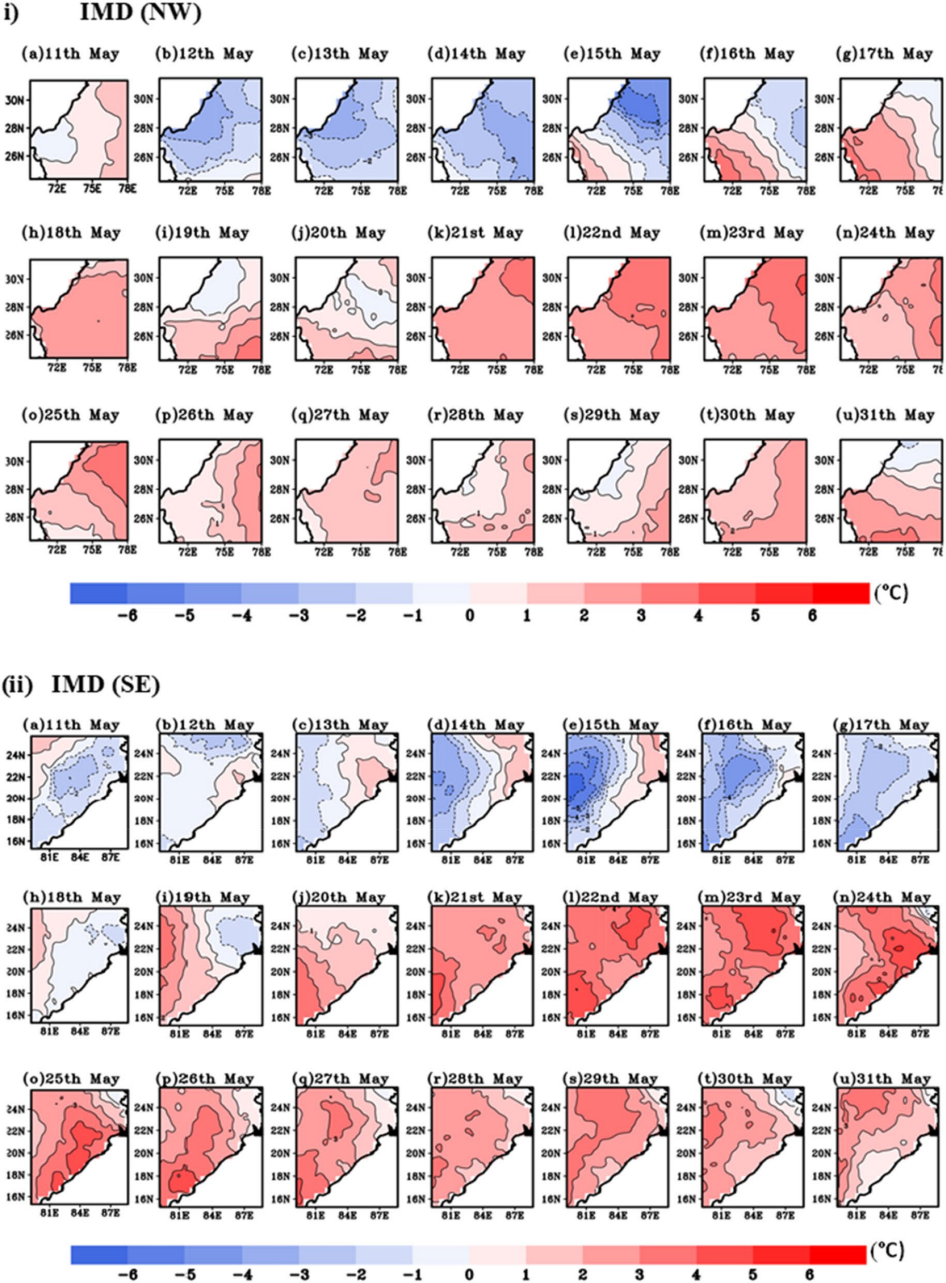

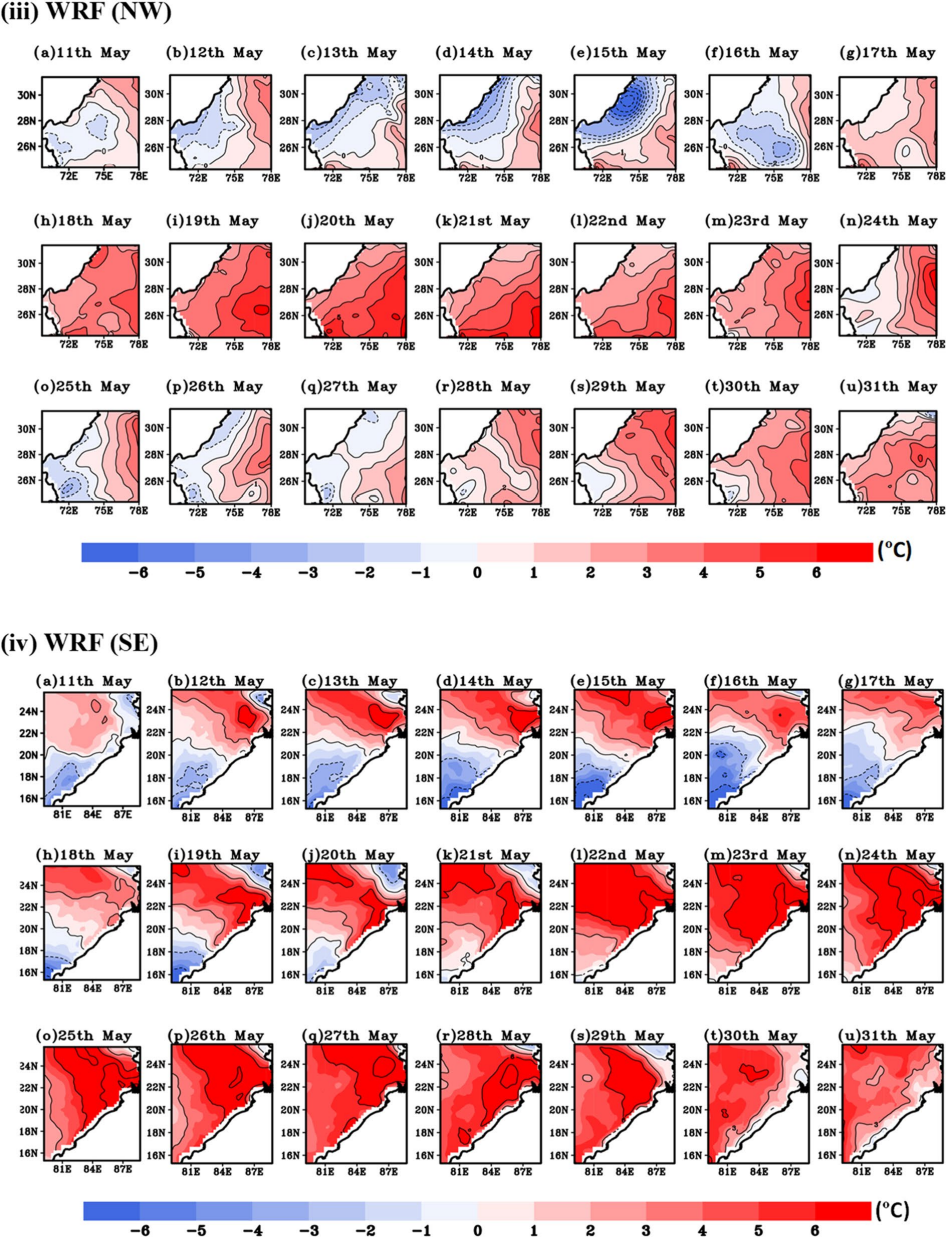
Extreme temperature anomalies (°C) based on (**i**), (**ii**) IMD and (**iii**), (**iv**) WRF simulated climatology for NW and SE region of India during 11th–31st May, 2015.

During summer 22nd to 28th May, 2015 north-western, central and eastern coastal region of India experienced severe HW condition leading loss of thousands human life in extreme high temperature condition^41^. Figure 8i,ii represents the observed and simulated temperature anomalies during the period of 11th to 31st May 2015. It clearly demonstrates the model ability to capture the high temperature over HW affected region i.e., NW and SE region. During 22nd to 30th May, HW events are identified with maximum temperature departure > 4.5 °C from the normal. Following IMD criteria, more than 4 °C temperature departure persists consecutive 3 days from IMD and WRF climatology confirm HW days over these regions. The simulated WRF and observed data captures HW days, since numerical models have their own uncertainties, it is lagging by 1–2 °C w.r.t IMD observed datasets.

##### Hovmoller plot analysing meteorological parameters during HW 2015:

**Figure 9:**
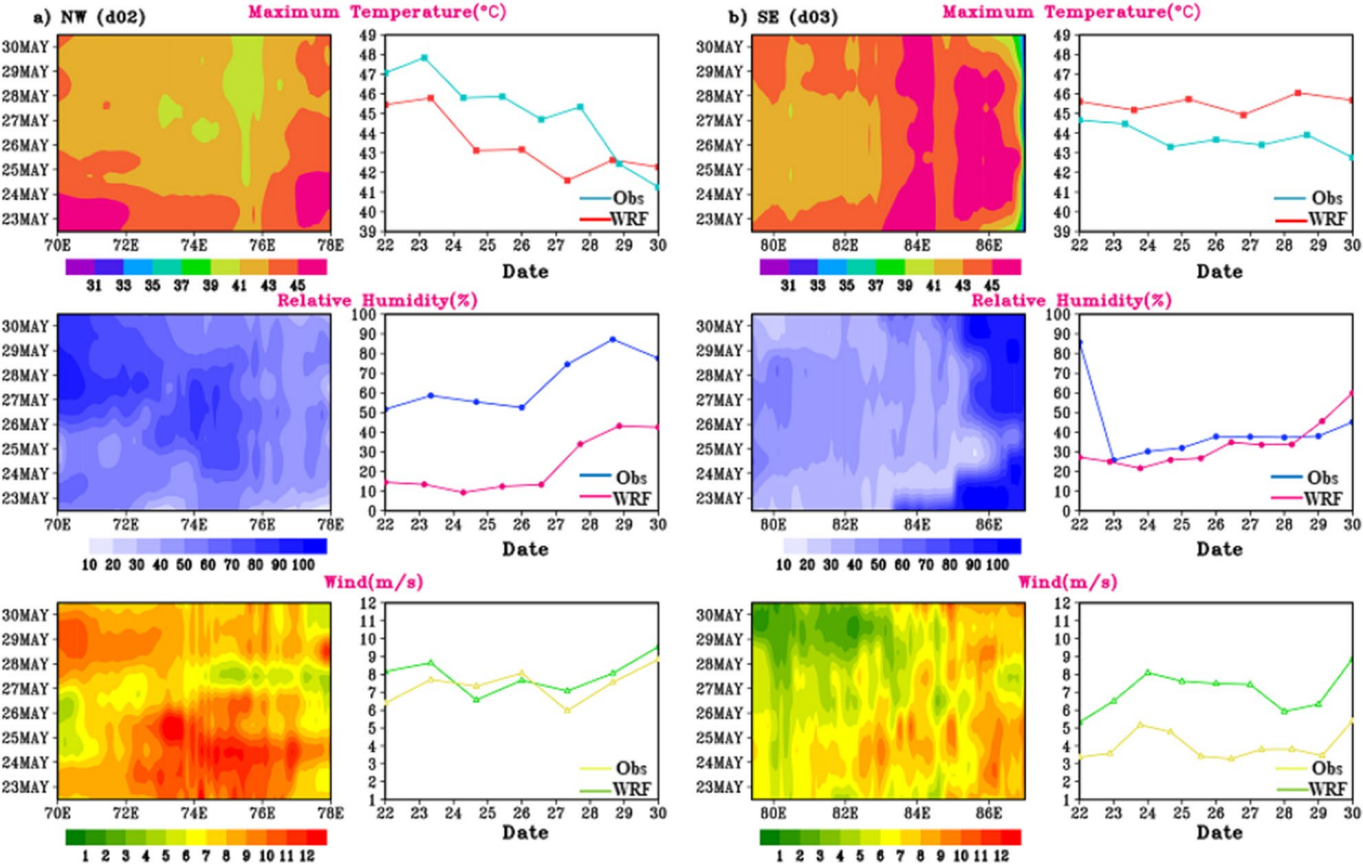
Longitudinal distribution of T_max_ (°C), RH (%) and wind magnitude (u_10_ & v_10_) on different days (y-axis) for the fixed latitude (27.8°N), and (20.8°N) of (**a**) d02 and (**b**) d03 domain respectively on spatial (left panels) temporal scale (right panels).

The simulated T_max_, RH and wind magnitude during HW days (22nd–30th May 2015) is analysed for d02 and d03 regions. Longitude-time plots averaged along fixed latitudes of 27.8° N (d02) & 20.8° N (d03) are shown in Fig. 9a,b it is inferred that in d02 region the spread of maximum temperature is in the range of 42–46 °C in spatial and temporal extent. A maximum temperature above 45 °C is reach on 22nd to 24th May, specially towards the 70–72° E and 78° E longitudinal regions. Moderate RH in range of 50–80% is observed in d02 region. While in d03 (Fig. 9b) i.e., SE region of India same analysis indicates a maximum temperature exceeding 46 °C, spread spatially and temporally along 83–85°E. it is seen that during 2015 HW case, SE region experienced a longer (8–9 days) period of maximum temperature with deficient amount of RH over entire d03 region, which resulted in heat wave over the region. As in^42^ mentioned that compared to other Indian region, south eastern India suffered a deadly 2015 heat wave with extreme temperature exceeded 45 °C in many places. As an additional diagnostic measure, the wind intensity has been calculated for both regions, revealing that the wind strength during episodes of extreme temperatures ranges from approximately 3 to 9 m/s in the d02 domain and 6 to 9 m/s in the d03 domain.

**Figure 10:**
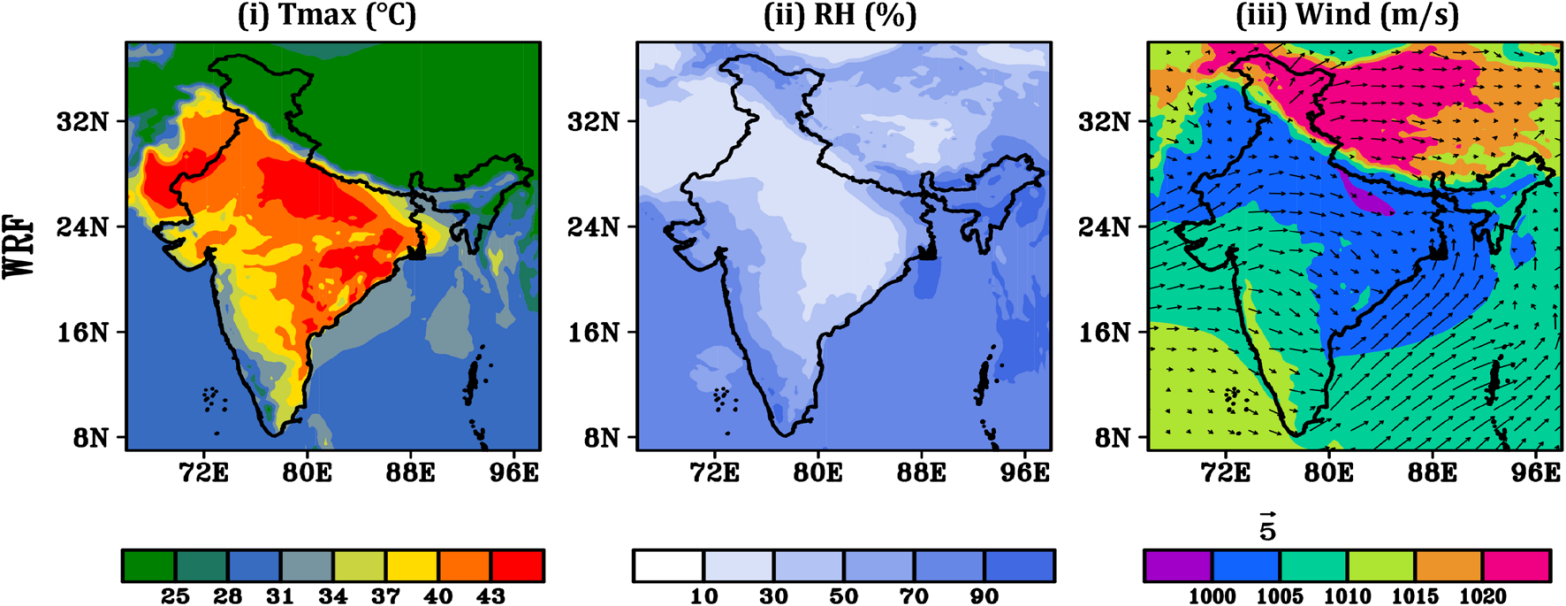
Model simulated dynamics of (**i**) T_max_, (**ii**) RH (**iii**) surface pressure and wind vector during HW days 2015 (22nd–30th May).

Further, to understand the dynamics behind rising HW intensity during HW days (22nd–30th May, 2015) influencing parameters like T_max_, RH and prevailing wind distribution have been studied across entire region of India. Globally, 2015 was the warmest year on record. Model simulated multiday maximum temperature reaches up to 45 °C over NW and SE coast of India. El-Nino effects, anthropogenic climate change, late monsoon etc. are the possible reason behind those days of higher temperature. Heat events over northern and mid-latitudinal continents are strongly linked to developing El Niña and La Niña events^43^. However, low temperature ranging 26 °C–32 °C is seen towards west coast region of India. It is clearly seen that in hilly region; especially western Himalaya, model is unable to capture the data perfectly. The simulated meteorological parameters are compared with the observed ERA5 datasets and the comparison (Supplementary Fig. 3) shows good agreement (− 1 °C to + 1 °C) between the model simulated and observations. Moreover, during 2015 HW event death toll appears to be higher than in other HWs during the same period across the globe. Relative humidity is one of the influencing factors behind maximum temperature rise. Figure 10ii shows low RH over HW affected region i.e., northwest, central and south east coastal India. Over the region, low RH in order of 20–40% is seen. Due to lower relative humidity (RH) levels, the daytime temperature rises rapidly in May, as the duration of daylight is longer. Consequently, the night time temperature is unable to cool significantly. These hot days have unusual atmospheric conditions with strong, warm, gusty dry winds blowing over the NW region of India. Whereas, SE India is surrounded by oceans it experiences higher temperature generally because of hot and humid conditions. The combination of high outgoing longwave radiation (OLR) and the absence of convection leads to a significant increase in maximum temperature. These episodes are often intensified by a persistent dry and hot wind. The model-simulated wind distribution pattern during extreme temperature week causing HW (22nd to 30th May 2015) are presented in Fig. 10iii.

Over India wind speed in range of 5 m/s is found during the HW episodes. These prevailing winds carry moisture from NW to interior regions of India. Dry air transport from the NW leading to moisture reduction along northwest and south east coast of India. It is also evident, westerly to north westerly winds majorly prevailed over most of the region of India with a cyclonic flow. Relative to SE India, NW India experienced strong and intense wind during the HW days.

#### Episodic event of 2016 HW

**Figure 11:**
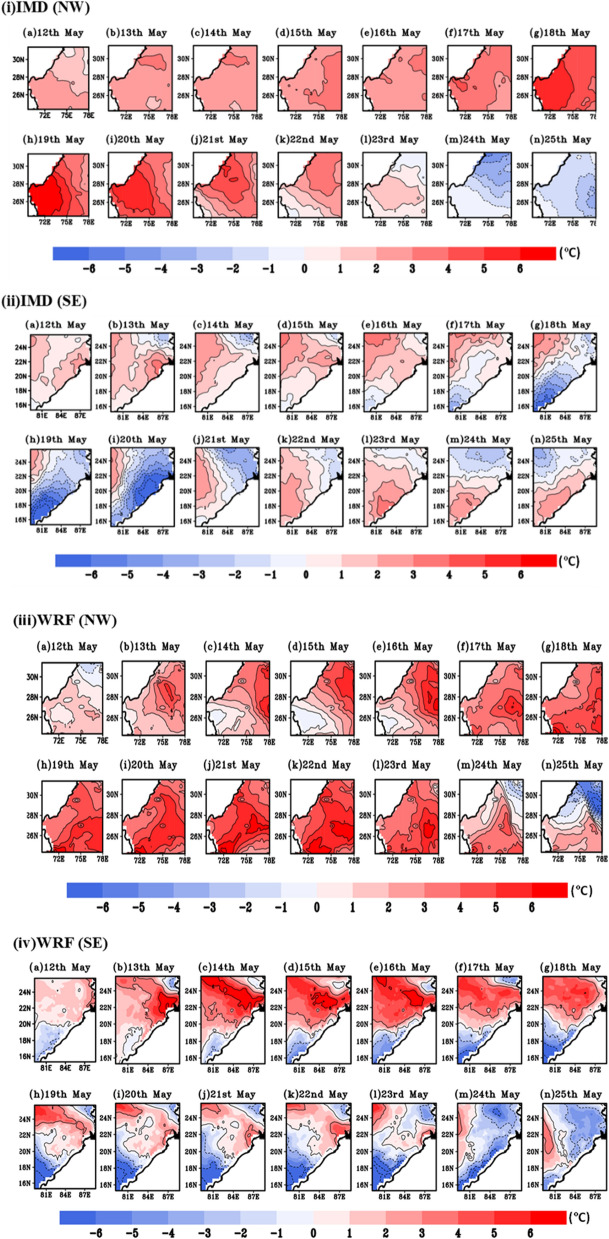
Similar to previous plot, extreme temperature anomalies representation based on (**i**), (**ii**) IMD and (**iii**), (**iv**) WRF simulated climatology for NW and SE region of India during 12th–25st May, 2016.

In order to further demonstrate the model reliability in predicting HW events, another case study of 2016 HW days over NW and SE regions of India at 9 km resolution are being simulated utilizing the same model configuration discussed in the preceding section. Similar to previous year (HW 2015), maximum temperature departure (12th–25th May, 2016) from IMD and WRF climatology is shown in Fig. 11i,ii,iii,iv respectively. Following IMD, HW criteria T_max_ departure more than 4.5 °C is observed during 14th to 23rd May. It is evident from the Fig. 11 that in year 2016, NW region is more severely affected by HW event than SE Indian region. A high temperature record over India is seen on May 19, 2016. In the Phalodi city of Rajasthan, the high temperature hit 51 °C. On that day, temperatures were high in most of the Rajasthan, with most stations recording maximum temperatures exceeding 46 °C.

##### Hovmoller plot analysing the meteorological parameters during HW 2016

**Figure 12:**
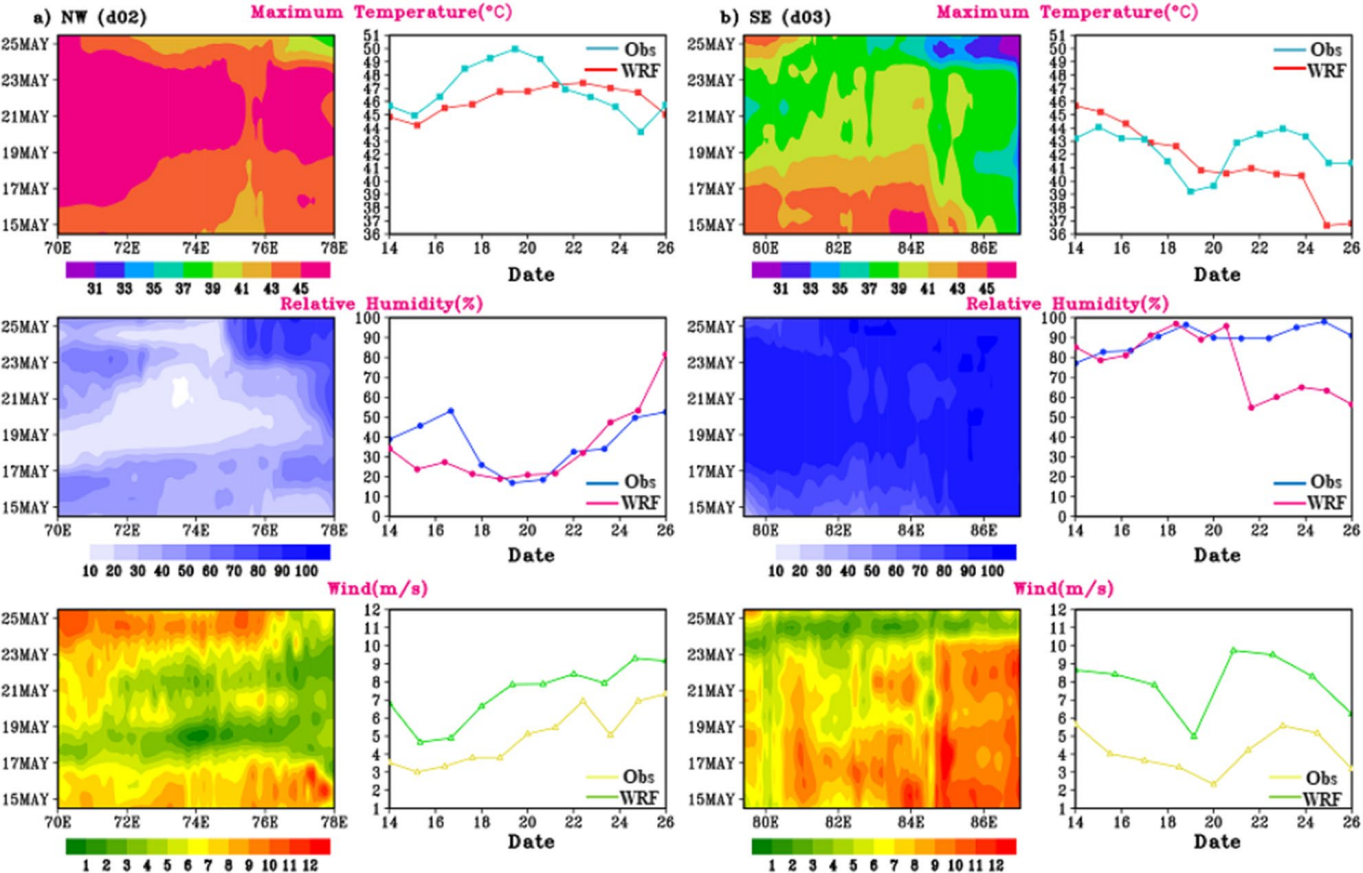
Time-longitude plot (Hovmoller representation) of T_max_ (°C), RH (%) and wind magnitude (u_10_ & v_10_) during 14th–26th May, 2016 HW days for (**a**) d02 and (**b**) d03 regions.

The Hovmoller plot for 14th to 26th May 2016 simulated T_max_, RH and wind magnitude during HW days are depicted in Fig. 12. The NW (d02) region recorded the hottest day from 16th to 22nd May with a maximum temperature reaches beyond 45 °C with moderate RH of 15–60%. May 19, 2016 recorded as the hottest day across the NW India, with temperatures reaching up to 48 °C before the cooling southwest monsoon rains arrive in July. While d03 recorded the moderate temperature ranging from 35 to 44 °C with high RH reaching around 60–99%. HW in North West and central India is commonly connected by unusual blocking across the North Atlantic Ocean. In the eastern coastal region of India, heatwaves (HW) are observed to coincide with westerly anomalies over the Indian landmass.

**Figure 13:**
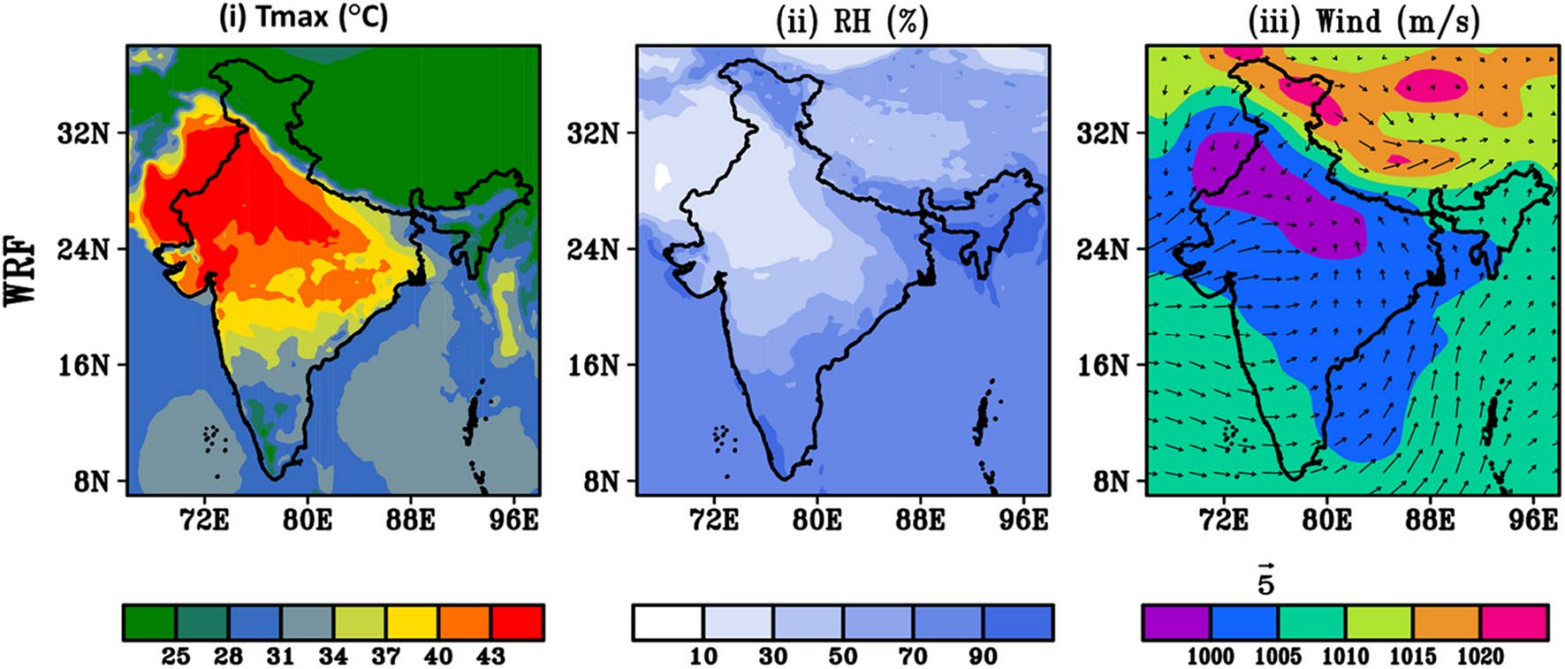
Same as previous plot model simulated dynamics of (**i**) T_max_ (°C), (**ii**) RH (%) (**iii**) surface pressure and wind vector during HW days 2016 (14th–23rd, May).

These anomalies weaken the land-sea breeze along the coastline, as noted by Ratnam et al.^42^. Consequently, hot and dry conditions prevail due to the presence of Loo, an intense afternoon overland wind that occurs across the India. Model simulated T_max_, RH, surface pressure and wind circulation patterns averaged over the HW period 14th to 23rd May, 2016 are shown in Fig. 13i–iii. T_max_ reaches up to 43 °C is soar towards NW regions of India, SE region is less affected in this year. There is clear humidity variation near the surface across the entire India. The RH in 2016 HW days is generally moderate towards NW region and higher towards SE region. This year appears to be normally very much humid in the summer hence the impact of most lethal HW is not solely attributed to high temperatures but also influenced by the effects of humidity. WRF simulation shows good agreement with the observed ERA5 (shown in Supplementary Fig. 4). Prevailing wind (≥ 5 m/s) with anti-clock wise wind direction is seen over major parts of the country, specially towards Bay of Bengal (BOB) and south eastern region of India. It may carry moisture from BOB region and pour heavy moisture towards eastern coastal region.

## Conclusions

In this study, WRF simulations of two extreme heat wave events (i.e., May 22nd–30th, 2015 and May 11th–26th, 2016) have been conducted at d01 (27 km), d02 (9 km) and d03 (9 km) regions. The present work examines the sensitivity with different combination of configuration for WRF simulations of extreme heat events over NW, SE and entire regions of India. The initial test discarded inadequate combination of parameterizations, keeping only six valid configurations for the sensitivity analysis. The combination of WSM6 microphysics (MP) along with radiation parameterization CAM, Yonsei (PBL), NOAH land surface, and Grell-3D convective schemes proves to be effective for heat wave events over the northwest and southeast regions of India. The WSM6 microphysics scheme accurately represents the cloud and precipitation process, ensuring proper simulation of convective activity and heat wave dynamics. The radiation parameterization CAM captures the radiative transfer processes and their impact on surface heating, enabling a realistic representation of heat wave conditions. The Yonsei planetary boundary layer (PBL) scheme accurately simulates the vertical mixing of heat and moisture, crucial for capturing the atmospheric stability and temperature profiles during HWs. The NOAH land surface accounts for soil moisture and vegetation dynamics, providing accurate surface temperature and energy fluxes. Lastly, the Grell-3D convective scheme effectively simulates deep convection, which is essential for capturing the intensity and spatial distribution of convective systems associated with HW events. Together, these combinations of scheme ensure a comprehensive representation of the key processes driving HW events, making it suitable for studying HWs in the NW and SE regions of India. Based on the robust statistics best combination of simulation is selected. The T_max_ variability during a HW event is well captured by Exp2 with a slight systematic temperature overestimation (+ 1 °C to + 2 °C), which is due to the complex interactions and uncertainties associated with soil moisture, evapotranspiration, vegetation dynamics, thermal diffusion, and model resolution.

Furthermore, to understand the possible dynamics during HW days, two case studies have been analysed; namely HW 2015 (22nd–30th May) and HW 2016 (14th–23rd May), and influencing parameters such as T_max_, RH, and prevailing wind distribution have been studied across the entire region of India. Model-simulated multiday mean maximum temperatures during HW 2015 reach upto 44 °C in NW and SE region of India. Strong El Nino effects, anthropogenic climate change, etc. might be possible causes of the temperature rise on those days. While, the episodic occurrence of HW 2016 has also been studied, and it shows that the second to third week of May was the warmest days, with temperatures reaching up to 40 °C to 45 °C, especially in the NW part of India. Those high temperature days have unusual atmospheric conditions with strong warm, gusty dry winds blowing over the NW region of India. These two case studies considered here illustrate that deadly heat waves are complicated phenomena, in aspects of both their physical characteristics and their impacts. There are numerous meteorological reasons for HW events to occur. HW in continental interiors are often associated with persistent high-pressure systems that lead the air to stagnate. In cases like the 2015 HWs, high temperatures are experienced, the temperature and humidity parameter reveal that it is the combination of the two fields that created a rare and deadly event during 2015 and 2016 HW case study. In SE region closer to the ocean, humidity played an important role. This is especially true in the 2015 HW event as daily maximum air temperatures were high with low RH. While in HW 2016 though temperature is low but high RH may cause a HW like phenomena. As in continental interiors, extreme temperatures will continue to increase as the overall climate warms, although perhaps at a somewhat lower rate. These episodic events were clearly distinct in terms of length, intensity, and geographic dispersion, which is well reflected by WRF model according to comparative studies. Thus, using the WRF model setup to predict heat wave conditions in advance can be very helpful for future forecast and can greatly aid in managing natural disasters, effects, and disaster mitigation.

### Supplementary Information

Supplementary Information.

## Data Availability

All the data sets used in this present study are available publicly and the same has been provided in the manuscript as well as in the acknowledgement section.
