# Supplementary material for: Fidelity of WRF model in simulating heat wave events over India
Source: Sci Rep. 2024 Feb 1;14:2693. doi: 10.1038/s41598-024-52541-2 (PMC10834968; doi:10.1038/s41598-024-52541-2)
Supplement: Supplementary file 1 — Supplementary Information. [file 41598_2024_52541_MOESM1_ESM.docx]

**Supplementary:**

***Fig.1.* *Daily averaged all Indian maximum temperature distribution during HW event duration, 22^nd^-30^th^ may 2015 (a) ERA5 data set (b) Exp1, (c) Exp2, (d) Exp3, (e) Exp4, (f) Exp5, and (g) Exp6.***

***Fig. 2 (a-f). Spatial distribution of maximum temperature bias (°C) of WRF w.r.t ERA5 datasets during HW event duration (22-30 may,2015).***

***Fig.3: Model simulated 2015 HW compare with ERA5 observed datasets.***

***Fig.4: Model simulated 2016 HW compare with ERA5 observed datasets.***
